# Biochemical Markers in Pleural Fluid for the Diagnosis of Tuberculous Pleural Effusion: A Retrospective Study

**DOI:** 10.3390/diseases14090327

**Published:** 2026-09-09

**Authors:** Natalia Zaporojan, Ramona Hodișan, Claudiu Zaporojan, Andreea Atena Zaha, Andrei Nicolae Csep, Dana Carmen Zaha

**Affiliations:** 1 Doctoral School of Biomedical Sciences, University of Oradea, University Street 1, 410087 Oradea, Romania; panus.natalia@student.uoradea.ro (N.Z.); zaporojan.claudiugheorghe@student.uoradea.ro (C.Z.); zaha.andreeaatena@student.uoradea.ro (A.A.Z.); dzaha@uoradea.ro (D.C.Z.); 2 Department of Preclinical Disciplines, Faculty of Medicine and Pharmacy, University of Oradea, 1 December Square 10, 410073 Oradea, Romania; 3 Department of Psycho-Neurosciences and Recovery, Faculty of Medicine and Pharmacy, University of Oradea, 1 December Square 10, 410073 Oradea, Romania; csep.andrei@uoradea.ro

**Keywords:** tuberculous pleural effusion, adenosine deaminase, lactate dehydrogenase, total protein, diagnostic accuracy

## Abstract

Background/Objectives: The rapid diagnosis of tuberculous pleural effusion (TPE) presents significant challenges for clinicians due to its paucibacillary nature. Routine biochemical markers may complement microbiological and molecular tests, especially in resource-limited settings. The objective of this study was to evaluate the diagnostic performance of pleural fluid adenosine deaminase (ADA), lactate dehydrogenase (LDH), total protein (TP), and the ADA/LDH and ADA/TP ratios for tuberculous pleural effusion, using Löwenstein–Jensen culture and Xpert MTB/RIF as microbiological reference standards Methods: We conducted a retrospective study (2016–2024) including 325 consecutive patients investigated for suspected TPE in a tertiary hospital. ADA, TP and LDH were measured, and additional ratio-based indices (ADA/TP, ADA/LDH) were calculated for 325 pleural fluid (PF) samples. Löwenstein–Jensen (LJ) culture (with MPT64 confirmation) and GeneXpert MTB/RIF (Xpert MTB/RIF) were used as separate microbiological reference standards. Sensitivity (Se), specificity (Sp), and areas under the ROC curve (AUC) were estimated, and optimal thresholds were determined using the Youden index. Results: Among 325 pleural fluid samples, 23 (7.1%) were culture-positive. Xpert MTB/RIF was performed in 73/325 patients (22.5%), with *Mycobacterium tuberculosis* detected in 19/73 (26.0%) tested samples. In PF, ADA provided the best performance against culture, with Se of 100%. Correlation analysis in PF showed a moderate positive association between TP and LDH (*p* < 0.0001). Conclusions: Among routine biochemical markers, PF ADA shows high NPV. Integrating these rapid, low-cost tests alongside LJ culture and Xpert MTB/RIF may streamline the diagnostic pathway for TPE.

## 1. Introduction

Tuberculosis (TB) continues to have a major impact on global public health: in 2023, an estimated 10.8 million new cases and approximately 1.25 million deaths were reported, despite the availability of modern diagnostic and treatment methods. In the 2024 Global TB Report, the disease is again listed among the leading causes of death from infection worldwide [1]. Extrapulmonary forms of disease (EPTB) generally account for 15–20% of all reported TB cases, with an even higher proportion among patients with immunosuppression or HIV co-infection [2,3].

In the EU/EEA, 19.0% of notified TB cases in 2022 were extrapulmonary, and Romania accounted for 25.6% of all notified cases and had the highest TB notification rate in the EU/EEA [4]. National data published in recent years indicate a decreasing trend in TB incidence in Romania compared with the early 2000s, but rates remain high in certain vulnerable groups. In this context, extra-respiratory forms, particularly pleural, osteoarticular, and lymph node tuberculosis, represent an important proportion of diagnosed cases in a share comparable to that described at international level [5,6].

Within the spectrum of EPTB, tuberculous pleural effusion (TPE) is one of the most important clinical sites and is associated with considerable morbidity and mortality. This form is usually paucibacillary and may present with nonspecific clinical manifestations, making early recognition and etiological confirmation difficult [3,7,8].

Under these circumstances, the evaluation of biochemical markers in body fluids, particularly adenosine deaminase (ADA) activity, lactate dehydrogenase (LDH) levels and total protein (TP) concentration may provide useful additional information that supports the diagnosis of TPE when interpreted together with clinical, imaging, and microbiological findings [7,9,10,11].

For TPE, pleural fluid (PF) ADA activity is one of the most extensively studied biomarkers. Numerous investigations have demonstrated its high diagnostic accuracy and its incorporation into diagnostic algorithms and predictive models, often in combination with other biochemical or inflammatory markers [9,12].

ADA is a key enzyme in purine metabolism, catalyzing the deamination of adenosine and deoxyadenosine. Its expression is particularly high in lymphoid tissue, especially in activated T lymphocytes, and it is essential for their normal development and function [13]. In PF, ADA is one of the most widely used biomarkers for tuberculous pleuritis, with numerous studies and systematic reviews confirming high diagnostic accuracy, sensitivities and specificities often exceeding 90% at commonly used cut-offs around 35–40 U/L [12,14].

More recently, the ADA2 isoenzyme has been shown to discriminate tuberculous from non-tuberculous pleural effusions with high accuracy, with sensitivity and specificity around 90%, although its measurement is not yet widely available in routine laboratories [15].

Conversely, the pleural fluid ADA/LDH ratio has been proposed as an additional marker to improve the etiological differentiation of effusions, particularly between tuberculous, parapneumonic, and malignant pleural effusions. However, results are heterogeneous: some studies suggest a small gain in diagnostic performance, whereas others do not show a clear advantage over ADA alone, with similar areas under the ROC curve [16,17].

Rapid molecular tests such as GeneXpert MTB/RIF (Xpert MTB/RIF) and GeneXpert MTB/RIF Ultra (Xpert MTB/RIF Ultra) have significantly shortened the time to diagnosis and, at the same time, allow detection of rifampicin resistance. Nevertheless, in paucibacillary specimens, particularly pleural fluid, the reported sensitivity remains only moderate despite very high specificity, so these assays are used in practice mainly as rule-in rather than rule-out tools [18,19].

More broadly, studies comparing conventional culture with molecular approaches have demonstrated the complementary value of these methods for the detection and identification of the *Mycobacterium tuberculosis* complex, with molecular techniques offering more rapid identification than conventional culture [20].

Although pleural fluid ADA levels generally have good negative predictive value for ruling out TPE, the literature also describes tuberculous pleural effusions with ADA levels below the diagnostic threshold that were later confirmed by culture and/or histopathology, underscoring the risk of rejecting the diagnosis on the basis of a single test [21,22].

Pleural fluid ADA has been extensively investigated for the diagnosis of tuberculous pleural effusion, the comparative diagnostic performance of routinely available biochemical markers and derived ratios, assessed against different microbiological reference standards, remains insufficiently characterized in our clinical setting.

In this context, we therefore hypothesized that pleural fluid ADA, alone or in combination with routinely available biochemical parameters such as TP and LDH, could provide useful diagnostic discrimination for tuberculous pleural effusion when evaluated against Löwenstein–Jensen culture (LJ culture) and Xpert MTB/RIF as separate microbiological reference standards.

## 2. Materials and Methods

### 2.1. Study Design

We conducted a single-center, observational, retrospective diagnostic accuracy study at the Bihor County Emergency Clinical Hospital (tertiary care center), Romania, over the period 2016–2024. A total of 325 patients evaluated for suspected tuberculous pleural effusion were included with pleural effusion (pleural fluid samples).

### 2.2. Study Population

We included adults and children evaluated for clinical suspicion of TPE for whom microbiological and biochemical investigations were requested on pleural fluid. Detailed inclusion and exclusion criteria are presented in Table 1.

### 2.3. Specimen Collection and Processing

Pleural fluid was obtained by imaging-guided (ultrasound) thoracentesis, with a volume of ~20 mL, into a sterile container. Samples were transported immediately to the laboratory. For microbiological investigation, PF was centrifuged (~3000× *g*, 10 min), and the sediment was inoculated into Löwenstein–Jensen medium and incubated at 37 °C for 6–8 weeks, with periodic readings according to standard procedures. PF samples were inoculated onto commercially prepared Löwenstein–Jensen medium for BK isolation, 8 mL, supplied in ready-to-use tubes, manufactured by the “Cantacuzino” National Institute for Medical-Military Research and Development, Bucharest, Romania. Identification of *Mycobacterium tuberculosis* complex isolates was performed using an MPT64 antigen detection test (SD BIOSENSOR, Cheongju-si, Republic of Korea). Molecular testing was performed using the Xpert MTB/RIF assay (Cepheid AB, Solna, Sweden) on the GeneXpert platform, according to the manufacturer’s instructions; when volume permitted, the sample was preconcentrated by centrifugation prior to cartridge loading.

### 2.4. Biochemical Determinations and Units of Measurement

In PF were measured: total protein (TP, g/dL), lactate dehydrogenase (LDH, U/L), and ADA (U/L). Analyses were performed on the Flexor Junior automated biochemistry analyzer (Vital Scientific N.V., Dieren, The Netherlands). ADA was measured using an BioSystems (Barcelona, Spain) reagents, and ELITechGroup (Puteaux, France) enzymatic kit, which includes reagents, two levels of internal quality controls, and a calibrator. The kit reagents are compatible with the instrument and the working method used. Internal quality control, at two levels, was run with each patient batch.

### 2.5. Reference Standards

The reference standards were matrix-specific LJ culture with MTBC confirmation by MPT64 and Xpert MTB/RIF performed on PF. The diagnostic accuracy of the index tests was assessed in separate analyses using LJ culture and Xpert MTB/RIF as reference standards. Culture results were recorded as positive/negative, while Xpert MTB/RIF results were recorded as detected/not detected. Clinical assessment (including clinical presentation and imaging findings) guided routine patient management; however, clinical criteria, histology, imaging, and follow-up were not incorporated into the reference standard for the primary diagnostic accuracy analyses. In addition, a standardized clinical category of “probable/possible” tuberculosis was not uniformly available in this retrospective dataset.

### 2.6. Case Definition

Case status was defined separately by specimen type and by the reference standard used in each analysis. For TPE, reference testing was performed on PF. Confirmed TPE was defined as a positive result by LJ culture and/or Xpert MTB/RIF. Non-TPE was defined as a negative result on the corresponding reference test used in that analysis (culture-negative for culture-based analyses; Xpert-not detected for Xpert-based analyses). ADA, total protein, and LDH were evaluated as index tests and were not used to define case status. A standardized “probable/possible” clinical TB category was not uniformly available in this retrospective dataset; therefore, primary analyses focused on microbiologically defined case status.

### 2.7. Statistical Analysis

Data analysis was performed using IBM SPSS Statistics (version 26.0) and the open-source software R (version 4.3.1). Data were summarized using tables and graphs. Continuous variables with a normal distribution were presented as mean (± standard deviation (sd)), whereas variables with non-normal distribution were expressed as median (interquartile range (IQR)). Comparisons of unpaired categorical variables were performed using the Chi-square test and Fisher’s exact test. Spearman’s correlation coefficient was used to assess the relationship between quantitative variables. To evaluate the performance of parameter values (TP, LDH, ADA, as well as ADA/LDH and ADA/TP), in comparison with culture and the molecular Xpert MTB/RIF test, Receiver Operating Characteristic (ROC) analysis and the Area Under the Curve (AUC) were performed. Sensitivity (Se) and specificity (Sp) were calculated based on the obtained cut-off values. Culture and Xpert MTB/RIF assay were considered dependent variables. Optimal thresholds were determined using the Youden index (sensitivity + specificity − 1). Confidence intervals (95% CI) for AUC were calculated using the DeLong asymptotic method, and in the case of small samples, bootstrapping with 2000 replicates. In addition, based on the optimal thresholds calculated by AUC, the performance of biochemical markers was evaluated in relation to culture and the Xpert MTB/RIF test by calculating the positive and negative predictive values (PPV and NPV), positive and negative likelihood ratios (LR+ and LR−) reported with 95% confidence intervals (95% CI). A multivariable logistic regression model was constructed to assess the predictive value of TP, LDH, and ADA in pleural fluid, using culture as the dependent variable. The model’s performance was verified by the Hosmer–Lemeshow test, and internal validation by bootstrap with a sample size of 1000. A *p*-value < 0.05 was considered statistically significant.

### 2.8. Ethical Considerations

This study was approved by the Ethics Committee of the Bihor County Emergency Clinical Hospital (approval code 11325, approval date 5 April 2024). All methods used in this study were performed in accordance with relevant guidelines and regulations. Each participant signed informed consent before undergoing examination.

## 3. Results

### 3.1. Patients Demographic Characteristics

**Figure 1:**
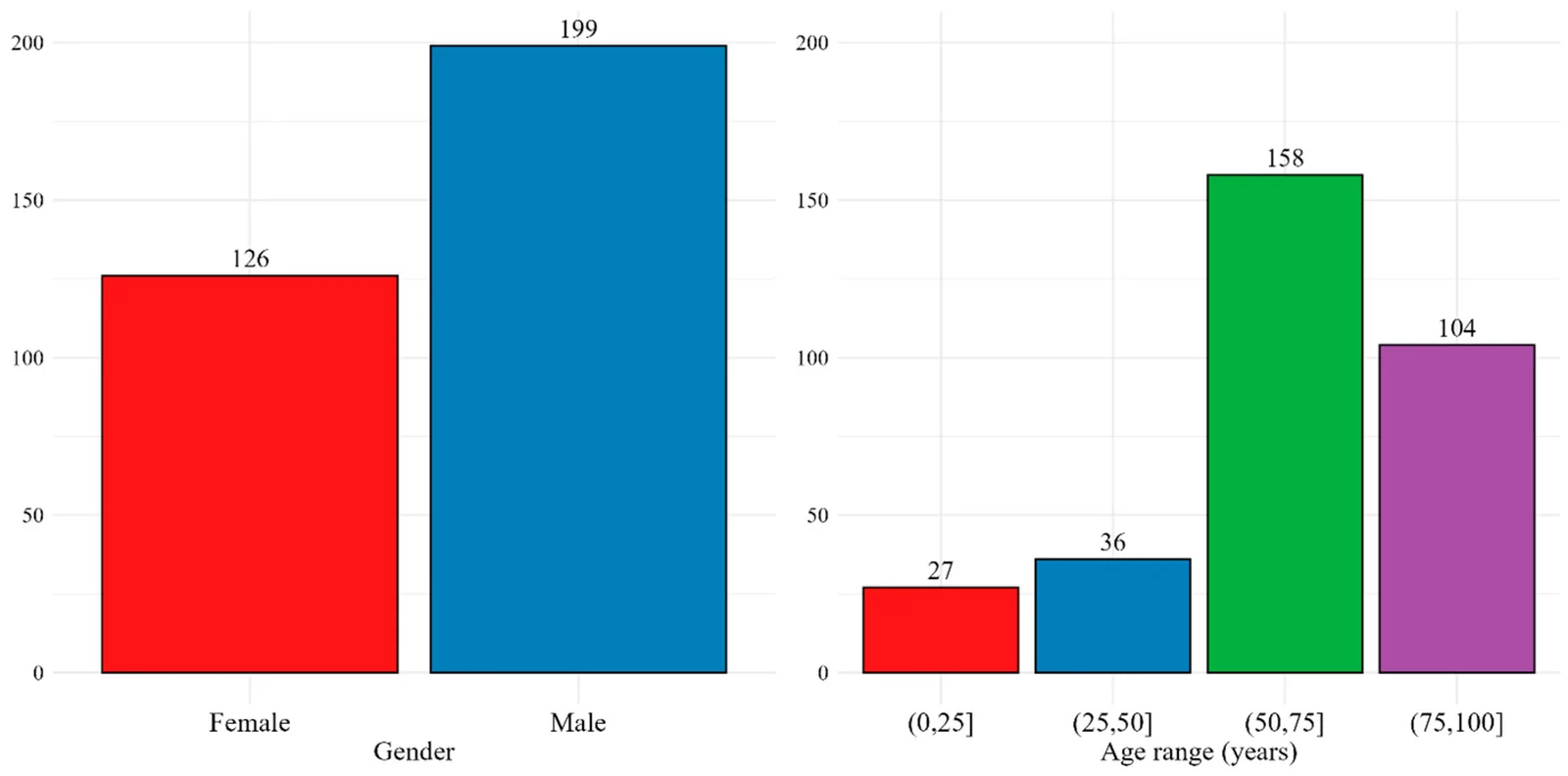
Distribution of patients by sex and age groups in the PF analysis.

In the PF study, 325 patients were analyzed. Patient age ranged from 1 to 97 years, with a median of 70 years (IQR 21). A male predominance was observed (61.23%), and most patients were older than 50 years (80.62%; age groups 51–75 and 76–100). The patients’ demographic characteristics are presented in Figure 1.

### 3.2. Biochemical Markers in Pleural Fluid

The biochemical markers analyzed were TP, LDH, and ADA in PF (Table 2). Patients with TP > 3 g/dL, LDH > 300 U/L, and ADA > 33 U/L, based on the prespecified thresholds, were classified as having values suggestive of risk for tuberculous pleural effusion. TP values ranged from 0.5 to 8.4 g/dL, with a median of 3.7 g/dL (IQR 1.7). LDH ranged from 27 to 10,000 U/L, with a median of 248 U/L (IQR 514). ADA values ranged from 1 to 150 U/L, with a median of 12 U/L (IQR 24).

In Table 3, the distribution of patients according to the biochemical marker cut-offs is presented.

At the prespecified cut-offs, the proportions of positive results were 66.76% for TP, 42.46% for LDH, and 22.46% for ADA. No significant differences were observed between women and men for any of the three markers (*p* > 0.05 in all three comparisons). In contrast, significant differences were found across age groups for all three markers (*p* < 0.001), with higher proportions above the cut-off among younger patients for TP > 3 g/dL: 100% in patients aged <25 years and 97.22% in those aged 26–50 years.

Table 4 presents the minimum, maximum, and median values of the ADA/LDH and ADA/TP ratios, as well as the correlation coefficient between these ratios.

The ADA/LDH ratio ranged from 0.001 to 0.291, whereas the ADA/TP ratio ranged from 0.323 to 36.585. All correlation coefficients indicated a moderate correlation with high statistical significance (*p* < 0.001 in all three comparisons), with the strongest association observed for ADA-LDH.

### 3.3. Performance Evaluation of TP, LDH, and ADA in Pleural Fluid

The performance of TP, LDH, and ADA was compared with culture and the molecular Xpert MTB/RIF assay, which were used as reference standards. In addition, the performance of the ADA/LDH and ADA/TP ratios was evaluated and compared with the two reference tests. A total of 325 cultures, of which 23 (7.1%) positive results and 302 negative results, respectively, and 73 Xpert MTB/RIF tests were performed, of which 19 (26.0%) presented with positive results (detected) and 54 with negative results (not detected). The results of the combined culture and Xpert MTB/RIF tests are presented in Table 5.

#### 3.3.1. Performance Evaluation of TP, LDH, and ADA Versus Culture in Pleural Fluid

**Figure 2:**
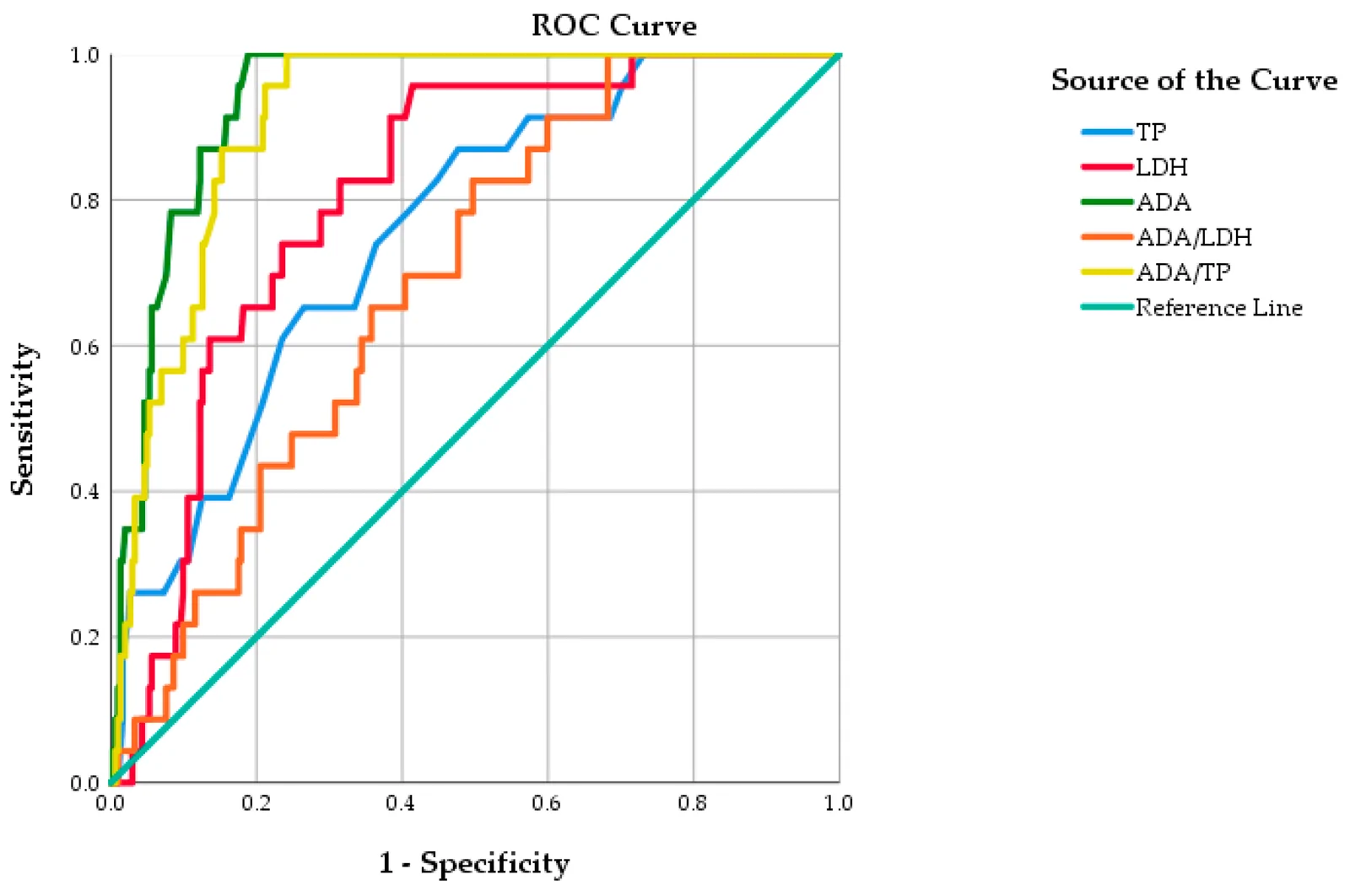
ROC analysis of TP, LDH, ADA, and the ADA/LDH and ADA/TP ratios versus culture (reference standard) in pleural fluid.

Table 6 presents the performance of TP, LDH, and ADA values, as well as the ADA/LDH and ADA/TP ratios, compared with culture in the pleural fluid analysis. Figure 2 shows the ROC analysis for the same parameters.

Among the markers evaluated against the reference standard (culture), ADA showed very good diagnostic performance, with an AUC of 0.938 (95% CI 0.909–0.967), 100% sensitivity and 81.10% specificity at the optimal cut-off of 29.5 U/L determined using Youden’s index, these aspects being in accordance with the specialized literature.

The AUC of 0.807 (95% CI 0.735–0.879) for LDH suggested very limited clinical utility, with a sensitivity of 95.7% and a specificity of 58.6% at a cut-off of 285 U/L. The weakest performance was observed for TP, with a sensitivity of 87%, a specificity of 52.3%, and an AUC of 0.756 (95% CI 0.665–0.847) at the optimal threshold of 3.65 g/dL. In all three evaluations, the AUC results were statistically significant (*p* < 0.05). The calculated indices, specifically the ADA/LDH and ADA/TP ratios, showed that ADA/TP had excellent diagnostic performance, with an AUC of 0.915 (95% CI 0.878–0.951).

Based on the optimal thresholds obtained through AUC analysis, the performance of the biochemical marker results (TP, LDH, and ADA values and the ADA/LDH and ADA/TP ratios) was evaluated in relation to culture, the reference standard, and the results are presented in Table 7. The highest LR+ values were observed for ADA, ADA/PT ratio and LDH (5.298, 4.137, and 2.311, respectively), suggesting good discrimination capacity.

#### 3.3.2. Performance Evaluation of TP, LDH, and ADA Versus Xpert MTB/RIF in Pleural Fluid

**Figure 3:**
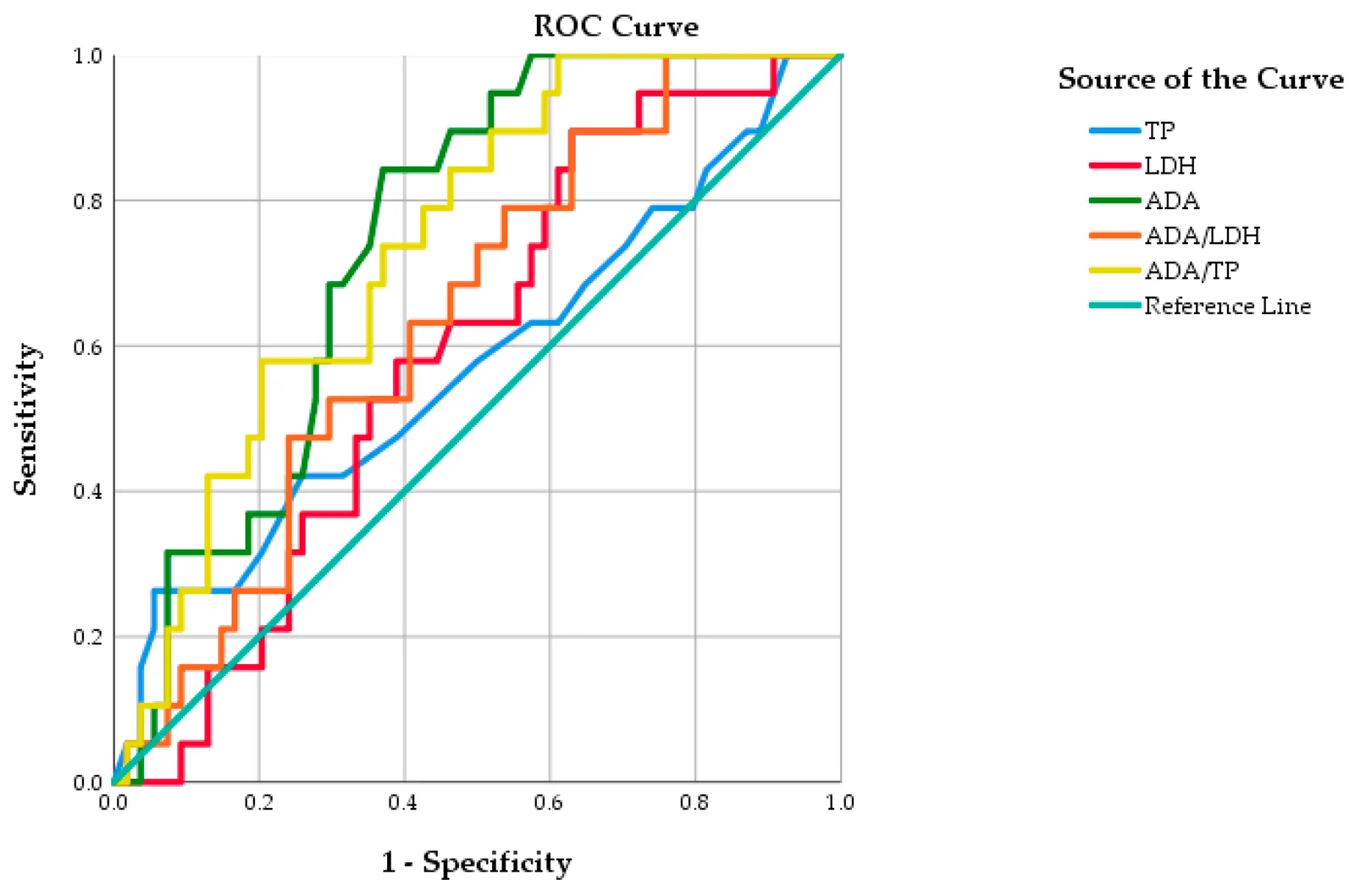
ROC analysis of TP, LDH, ADA, and the ADA/LDH and ADA/TP ratios versus Xpert MTB/RIF (reference standard) in the pleural fluid analysis.

Table 8 summarizes the performance of TP, LDH, and ADA values and the ADA/LDH and ADA/TP ratios compared with Xpert MTB/RIF in the pleural fluid analysis, while Figure 3 presents the corresponding ROC analysis for the same parameters.

In the performance analysis of the three markers compared with the molecular Xpert MTB/RIF assay, ADA showed the best performance, with a sensitivity of 84.20% and a specificity of 63% at the optimal cut-off of 44.5 U/L. The AUC-based performance of TP and LDH did not reach statistical significance (*p* = 0.361 and *p* = 0.183, respectively). The sensitivity of TP was 26.30% at an optimal threshold of 5.15 g/dL, while LDH showed a sensitivity of 89.50% at a threshold of 391 U/L. The AUC values for TP and LDH, 0.574 (95% CI 0.416–0.732) and 0.592 (95% CI 0.456–0.728), respectively, indicated poor performance.

By contrast, the ADA/LDH and ADA/TP ratios also showed limited performance, with AUC values of 0.639 (95% CI 0.506–0.773) and 0.739 (95% CI 0.622–0.855), respectively. The optimal Youden cut-offs were 0.0335 for ADA/LDH and 6.2842 for ADA/TP.

In pleural fluid, ADA was the best-performing individual marker versus culture, while ADA/TP was the best-performing calculated index. When compared with Xpert MTB/RIF, ADA remained clinically useful, and ADA/TP and ADA/LDH provided moderate diagnostic performance. These findings support the use of ADA (and ADA/TP) as complementary indicators in pleural fluid evaluation, with interpretation anchored in reference standards and pretest probability.

Based on the optimal thresholds obtained through AUC analysis, the performance of the biochemical marker results (TP, LDH, and ADA values and the ADA/LDH and ADA/TP ratios) was evaluated in relation to Xpert MTB/RIF, the reference standard, and the results are presented in Table 9. In the analysis of the performance of biochemical markers in relation to Xpert MTB/RIF, the highest LR+ values were observed for TP and ADA (4.737 and 2.274, respectively), showing good discrimination capacity.

#### 3.3.3. Multivariable Logistic Regression Model for Evaluating the Performance of TP, LDH, and ADA in Pleural Fluid

We then assessed the diagnostic performance of TP, LDH, and ADA in pleural fluid using a multivariable logistic regression model (Table 10). A total of 325 tests were included in the model, of which 23 tests had a positive result in culture, leading to a number of approximately seven events per variable. LDH and ADA were significant predictors of the condition (*p* < 0.05 for both), whereas TP did not show a significant effect (*p* = 0.126). Statistical significance was observed in the model calibration according to the Hosmer–Lemeshow test (*p* = 0.648). After bootstrap validation, ADA and LDH remained significant predictors (*p* < 0.01, 95% CI 0.043–0.107, and *p* = 0.035, 95% CI −0.02–0.000, respectively), while TP was not a statistically significant predictor (*p* = 0.118, 95% CI −0.262–1.156).

## 4. Discussion

In this study, we evaluated the diagnostic value of the biochemical markers ADA, LDH, and total protein measured in pleural fluid. In PF, we additionally calculated ratio-based indices derived from these markers (particularly ADA/TP and ADA/LDH). LJ culture and the Xpert MTB/RIF assay were used as microbiological reference standards. An important feature of our study is the evaluation of PF samples using a panel of biochemical markers and two reference standards, allowing a direct comparison of the clinical utility of these markers in tuberculous pleural effusion, and providing relevant information for resource-limited settings where microbiological methods may be less accessible or associated with longer turnaround times.

In pleural fluid, ADA showed the best diagnostic performance among the analyzed markers when culture was used as the reference standard, with an AUC of 0.938 and an optimal cut-off of >29.5 U/L, associated with 100% sensitivity and 81.1% specificity. In our cohort, no culture-confirmed tuberculous pleural effusion cases had ADA values below this threshold, while the proportion of false-positive results remained acceptable. When Xpert MTB/RIF was used as the reference standard, ADA demonstrated only moderate discriminatory ability (AUC 0.747, cut-off > 44.5 U/L), which may reflect both the lower sensitivity of molecular testing in paucibacillary pleural effusions and differences in the definition of a “positive case” between culture and molecular testing. Among the calculated ratio indices, ADA/TP showed the best agreement with culture (AUC 0.915), suggesting a potential role in adjusting ADA values for variations related to the protein concentration of the effusion. The complementary role of conventional culture and molecular methods for the identification of the *Mycobacterium tuberculosis* complex has also been demonstrated in other settings. Although the study by Neeraja et al. was conducted in bovines, it provides additional microbiological evidence supporting the combined use of culture and molecular approaches for *Mycobacterium tuberculosis* complex identification [20].

Overall, these results support the use of ADA as a first-line biochemical marker in the evaluation of pleural effusions suspected of tuberculosis. Our pleural fluid ADA results are consistent with recent meta-analyses and contemporary reviews/guidelines showing high diagnostic accuracy for TPE, with pragmatic thresholds in the 30–40 U/L range across different populations and settings [7,9,23]. In these series, ADA values > 70 U/L make TPE highly likely, whereas values < 40 U/L render the diagnosis unlikely in the context of a lymphocytic exudate, which is in line with the thresholds identified in our study when culture or Xpert MTB/RIF are used as reference standards. Compared with interferon-γ, ADA generally shows slightly lower overall diagnostic accuracy; however, the difference appears modest, and ADA remains preferred in routine practice due to its lower cost and broad availability [24]. Studies on ADA isoenzymes and recent reviews suggest that ADA2 accounts for most of the increased total ADA activity in TPE, although isoenzyme testing is not routinely available and remains largely confined to specialized centers [7,15,25]. Age and comorbidities may modulate ADA levels. Some studies suggest lower ADA values with increasing age and recommend caution, including consideration of pleural biopsy, when ADA falls in the intermediate range of 40–70 U/L, particularly in older patients [26,27]. Very high ADA values (≥250 U/L) are more frequently reported in empyema, complicated parapneumonic effusions, or lymphoma, underscoring that elevated ADA is not synonymous with TPE and must be interpreted in clinical and imaging context [28,29].

Recent guidelines, including the British Thoracic Society recommendations, incorporate ADA in the diagnostic approach to unilateral pleural effusions, particularly for lymphocyte-predominant exudates in high-prevalence settings, alongside microbiological and histological testing, supporting its role as a first-line adjunct in suspected TPE [23]. In parallel, molecular assays (Xpert MTB/RIF and Xpert MTB/RIF Ultra) on pleural fluid show modest sensitivity compared with other specimen types; although Ultra outperforms Xpert MTB/RIF, overall accuracy remains lower than that reported for ADA, positioning Ultra as a complementary tool rather than a replacement for biochemical markers [19]. Other pleural biomarkers, such as interleukins (IL-27), have shown promising results in some studies; however, they have not displaced ADA as a practical routine test, and evidence remains heterogeneous [30,31]. Regarding pleural effusions, our data confirm ADA as a first-line test in the evaluation of tuberculous pleural effusion. A threshold around 30–40 U/L remains pragmatic and aligned with existing meta-analyses, both for making TPE unlikely at low values and for supporting the diagnosis when ADA is clearly elevated [9,32].

Large meta-analyses of pleural fluid ADA commonly report pooled optimal cut-offs in the ~35–40 U/L range. In our cohort, the ROC-derived PF ADA threshold versus LJ culture was >29.5 U/L, slightly below this commonly reported range, whereas the threshold versus Xpert MTB/RIF was >44.5 U/L, higher than pooled estimates. These differences may reflect variation in reference standards, the smaller subset undergoing Xpert testing, and the paucibacillary spectrum, highlighting that apparent “optimal” cut-offs can shift with diagnostic pathways and case-mix. In our cohort, an ADA cut-off of 29.5 U/L ensured maximal sensitivity when culture was the reference standard, while a higher cut-off of 44.5 U/L provided the best sensitivity-specificity balance (Youden index) versus Xpert MTB/RIF. These observations suggest that ADA values should be interpreted cautiously, considering the reference standard used, local TB prevalence, and patient-specific characteristics [7,9,10,33,34]. Contextual interpretation remains essential because pleural ADA may be falsely elevated in empyema, complicated parapneumonic effusions, rheumatoid pleuritis, or certain malignancies, including lymphoma; very high values (>250 U/L) are more suggestive of empyema or lymphoma than TPE. In such situations, integrating clinical, imaging, and histological data is mandatory, and ADA should not be used in isolation for therapeutic decisions [28,35,36,37].

A strength of this study is the evaluation of pleural fluid using a biochemical panel (ADA, LDH, TP) and predefined ratio-based indices (ADA/TP and ADA/LDH). This approach allows a direct comparison of the clinical utility of these markers in a major and frequently encountered form of TPE in routine practice. Another advantage is the use of two reference standards, Löwenstein–Jensen culture and the Xpert MTB/RIF assay, which provides a more nuanced view of index test performance and shows how optimal cut-offs may vary depending on the chosen reference. In addition, the analyzed markers are widely available routine laboratory tests, including in resource-limited settings, which enhances the practical applicability of our findings.

However, these results should be interpreted in light of several limitations. First, the retrospective design relies on data recorded in routine practice, with the potential for incomplete information and case selection bias. Second, both culture and Xpert MTB/RIF are imperfect reference standards in paucibacillary infections; some “negative” cases may in fact represent tuberculosis, which could lead to underestimation or overestimation of the performance of the biochemical markers. Moreover, this was a single-center study conducted in a high TB-prevalence area; therefore, generalization to other populations, including lower incidence settings, and populations with different age or comorbidity structures, should be made cautiously. Finally, we did not include other potentially relevant biomarkers (IFN-γ, IL-27, or ADA isoenzymes), precluding a direct comparison with modern biomarker panels proposed in the literature.

Based on these findings, prospective studies, ideally multi-center, are needed to validate the proposed cut-offs for ADA, TP, and LDH in pleural fluid in independent cohorts, including populations with lower TB prevalence and different comorbidity profiles such as older adults, immunocompromised patients, and HIV co-infection. Integrating biochemical markers into a clinical score that incorporates clinical, imaging, and laboratory data, and potentially molecular testing, could improve pretest probability estimation and provide front-line clinicians with simpler decision-support tools. Another important direction is the evaluation of combined algorithms using ADA as a triage test (“rule in/rule out”) and Xpert MTB/RIF/Xpert MTB/RIF Ultra as confirmatory tests, supported by cost-effectiveness analyses across different prevalence and resource settings. In addition, exploring the role of ADA2 and other inflammatory or cytokine biomarkers in combination with the markers assessed in this study may further improve the diagnostic accuracy for tuberculous pleural effusion.

## 5. Conclusions

The main findings can be summarized as follows. In PF, ADA remained the biochemical marker with the best accuracy for tuberculous pleural effusion, with cut-offs consistent with the literature, while the calculated PF indices (especially ADA/TP) provided only a modest additional gain over ADA alone. These findings should be interpreted in light of the retrospective, single-center design of the study and the limited sensitivity of Löwenstein–Jensen culture and Xpert MTB/RIF in paucibacillary pleural tuberculosis, which may have resulted in misclassification of some microbiologically negative cases.

As a conclusion, routine biochemical markers, particularly pleural ADA activity provide important diagnostic value in tuberculous pleural effusion. In resource-limited healthcare settings, these tests may support early triage and prompt initiation of anti-tuberculosis therapy, complementing rather than replacing conventional and molecular microbiological methods.

## Figures and Tables

**Table 1:** Patient selection criteria.

Inclusion Criteria	Exclusion Criteria
• Clinical suspicion of pleural TPE. • Availability of at least one reference microbiological test: LJ culture and/or Xpert MTB/RIF. • Availability of the corresponding biochemical analyses performed on PF. • Complete paired records including both biochemical tests and the reference standard results.	• Insufficient sample volume for the planned tests. • Compromised samples (unidentified/mislabeled specimens, evident contamination, or insufficient material for all investigations). • Invalid/indeterminate results on Xpert MTB/RIF or reference tests that were not repeated. • Missing minimum data required to construct 2 × 2 tables (index test vs. reference standard).

**Table 2:** Descriptive statistics of biochemical markers in PF.

Parameter	TP (g/dL)			LDH (U/L)			ADA (U/L)		
	≤3 n = 108	>3 n = 217	Total n = 325	≤300 n = 187	>300 n = 138	Total n = 325	≤33 n = 252	>33 n = 73	Total n = 325
Minimum	0.5	3.1	0.5	27	303	27	1	34	1
Maximum	3	8.4	8.4	300	10,000	10,000	33	150	150
Median (IQR)	2.15 (1.12)	4.2 (0.90)	3.7 (1.7)	130 (101.5)	688 (872)	248 (514)	8 (9)	47 (26)	12 (24)

**Table 3:** Distribution of patients by sex and age according to pleural fluid TP, LDH, and ADA values.

Variable	TP (g/dL)		*p*-Value	LDH (U/L)		*p*-Value	ADA (U/L)		*p*-Value
	≤3	>3		≤300	>300		≤33	>33	
**Gender**									
Female	43	83	0.785	78	48	0.205	104	22	0.086
Male	65	134		109	90		148	51	
**Age**									
(0, 25]	0	27	*p* < 0.001	6	21	*p* < 0.001	6	21	*p* < 0.001
(25, 50]	1	35		7	29		17	19	
(50, 75]	53	105		102	56		137	21	
(75, 100]	54	50		72	32		92	12	

**Table 4:** ADA/LDH, and ADA/TP ratios and correlations between biomarkers in PF.

Ratio	Minimum	Maximum	Median (IQR)	Correlation	rho	*p*-Value
ADA/LDH	0.001	0.291	0.045 (0.053)	ADA and LDH	0.686	*p* < 0.001
ADA/TP	0.323	36.585	3.478 (5.748)	ADA and TP	0.593	*p* < 0.001

ADA = Adenosine deaminase, measured in U/L, LDH = Lactate dehydrogenase, measured in U/L, TP = Total protein, measured in g/dL.

**Table 5:** Number of combined culture and Xpert MTB/RIF tests performed in PF.

Xpert MTB/RIF	Culture		Total
	Positive	Negative	
Detected	17	2	19
Not detected	5	49	54
Total	22	51	73

**Table 6:** Performance of TP, LDH, and ADA values and the ADA/LDH and ADA/TP ratios compared with culture in the pleural fluid analysis.

	AUC (95% CI)	*p*-Value	Cut-Off (95% CI)	Se (% 95% CI)	Sp (% 95% CI)	Youden’s Index
TP vs. culture	0.756 (0.665–0.847)	*p* < 0.001	>3.65 (3.35–4.65)	87.00 (0.664–0.972)	52.30 (0.465–0.581)	0.393
LDH vs. culture	0.807 (0.735–0.879)	*p* < 0.001	>285 (284.5–981.5)	95.70 (0.781–0.999)	58.60 (0.528–0.642)	0.543
ADA vs. culture	0.938 (0.909–0.967)	*p* < 0.001	>29.5 (29–44.5)	100.00 (0.852–1.000)	81.10 (0.762–0.854)	0.811
ADA/LDH vs. culture	0.688 (0.598–0.779)	*p* < 0.001	>0.0429 (0.0259–0.0845)	82.60 (0.612–0.950)	50.30 (0.445–0.561)	0.329
ADA/TP vs. culture	0.915 (0.878–0.951)	*p* < 0.001	>6.4550 (6.366–9.884)	100.00 (0.852–1.000)	75.80 (0.706–0.805)	0.758

ADA = Adenosine deaminase, measured in U/L, LDH = Lactate dehydrogenase, measured in U/L, TP = Total protein, measured in g/dL.

**Table 7:** Performance of biochemical markers based on optimal thresholds obtained by AUC analysis compared with culture in pleural fluid analysis.

	Culture		PPV (95% CI)	NPV (95% CI)	LR+ (95% CI)	LR− (95% CI)
	Positive	Negative				
**TP**						
>3.65	20	144	12.2% (0.076, 0.182)	98.1% (0.947, 0.996)	1.824 (1.497, 2.222)	0.249 (0.086, 0.720)
≤3.65	3	158				
**LDH**						
>285	22	125	15% (0.096, 0.218)	99.4% (0.969, 1.000)	2.311 (1.969, 2.712)	0.074 (0.011, 0.506)
≤285	1	177				
**ADA**						
>29.5	23	57	28.7% (0.192, 0.400)	100% (0.985, 1.000)	5.298 (4.194, 6.694)	0.000 (0.000, −)
≤29.5	0	245				
**ADA/LDH**						
>0.0429	19	150	11.2% (0.069, 0.170)	97.4% (0.936, 0.993)	1.663 (1.336, 2.071)	0.346 (0.141, 0.848)
≤0.0429	4	152				
**ADA/TP**						
>6.4550	23	73	24% (0.158, 0.337)	100% (0.984, 1.000)	4.137 (3.388, 5.052)	0.000 (0.000, −)
≤6.4550	0	229				

ADA = Adenosine deaminase, measured in U/L, LDH = Lactate dehydrogenase, measured in U/L, TP = Total protein, measured in g/dL.

**Table 8:** Performance of TP, LDH, and ADA values and the ADA/LDH and ADA/TP ratios compared with Xpert MTB/RIF in the pleural fluid analysis.

	AUC (95% CI)	*p*-Value	Cut-Off (95% CI)	Se (% 95% CI)	Sp (% 95% CI)	Youden’s Index
TP vs. Xpert MTB/RIF	0.574 (0.416–0.732)	0.361	>5.15 (2.65–5.35)	26.30 (0.091–0.512)	94.40 (0.846–0.988)	0.208
LDH vs. Xpert MTB/RIF	0.592 (0.456–0.728)	0.183	>391 (262–1244)	89.50 (0.669–0.987)	37.00 (0.243–0.513)	0.265
ADA vs. Xpert MTB/RIF	0.747 (0.635–0.858)	*p* < 0.001	>44.5 (28.5–50)	84.20 (0.604–0.966)	63.00 (0.487–0.757)	0.472
ADA/LDH vs. Xpert MTB/RIF	0.639 (0.506–0.773)	0.040	>0.0335 (0.0253–0.0852)	89.50 (0.669–0.987)	37.00 (0.243–0.513)	0.265
ADA/TP vs. Xpert MTB/RIF	0.739 (0.622–0.855)	*p* < 0.001	>6.2842 (5.941–15.640)	100.00 (0.824–1.000)	38.90 (0.259–0.531)	0.389

ADA = Adenosine deaminase, measured in U/L, LDH = Lactate dehydrogenase, measured in U/L, TP = Total protein, measured in g/dL.

**Table 9:** Performance of biochemical markers based on optimal thresholds obtained by AUC analysis compared with Xpert MTB/RIF in pleural fluid analysis.

	Xpert MTB/RIF		PPV (95% CI)	NPV (95% CI)	LR+ (95% CI)	LR− (95% CI)
	Detected	Not Detected				
**TP**						
>5.15	5	3	62.5% (0.245, 0.915)	78.5% (0.665, 0.877)	4.737 (1.250, 17.954)	0.780 (0.592, 1.029)
≤5.15	14	51				
**LDH**						
>391	17	34	33.3% (0.208, 0.479)	90.9% (0.708, 0.989)	1.421 (1.100, 1.836)	0.284 (0.073, 1.103)
≤391	2	20				
**ADA**						
>44.5	16	20	44.4% (0.279, 0.619)	91.9% (0.781, 0.983)	2.274 (1.526, 3.387)	0.251 (0.087, 0.723)
≤44.5	3	34				
**ADA/LDH**						
>0.0335	17	34	33.3% (0.208, 0.479)	90.9% (0.708, 0.989)	1.421 (1.100, 1.836)	0.284 (0.073, 1.103)
≤0.0335	2	20				
**ADA/TP**						
>6.2842	19	33	36.5% (0.236, 0.510)	100% (0.839, 1.000)	1.636 (1.323, 2.024)	0.000 (0.000, −)
≤6.2842	0	21				

ADA = Adenosine deaminase, measured in U/L, LDH = Lactate dehydrogenase, measured in U/L, TP = Total protein, measured in g/dL.

**Table 10:** Multivariable logistic regression analysis of TP, LDH, and ADA in pleural fluid.

	β	SE	*p*-Value	OR (95% CI)
TP	0.439	0.288	0.126	1.552 (0.883–2.727)
LDH	−0.001	0	0.049	0.999 (0.999–1)
ADA	0.063	0.012	<0.001	1.065 (1.04–1.09)

*β* = regression coefficient; SE = standard error; OR = odds ratio; CI = confidence interval, ADA measured in U/L, LDH measured in U/L, TP measured in g/dL.

## Data Availability

The data presented in this study are available on request from the corresponding author due to privacy.

## Abbreviations

The following abbreviations are used in this manuscript:

ADA	Adenosine deaminase
AUC	Area under the curve
EPTB	Extrapulmonary tuberculosis
LDH	Lactate dehydrogenase
LJ culture	Löwenstein–Jensen culture
PF	Pleural fluid
ROC	Receiver operating characteristic
Se	Sensitivity
Sp	Specificity
TB	Tuberculosis
TBM	Tuberculous meningitis
TP	Total protein
TPE	Tuberculous pleural effusion
Xpert MTB/RIF	GeneXpert MTB/RIF
